# Laboratory Cultivation of *Vairimorpha (Nosema) ceranae* (Microsporidia: Nosematidae) in Artificially Infected Worker Bees

**DOI:** 10.3390/insects13121092

**Published:** 2022-11-28

**Authors:** Anastasia N. Ignatieva, Sergey A. Timofeev, Yuri S. Tokarev, Viacheslav V. Dolgikh

**Affiliations:** All-Russian Institute of Plant Protection, Podbelskogo 3, 196608 St. Petersburg, Russia

**Keywords:** *Nosema ceranae*, *Vairimorpha ceranae*, *Apis mellifera*, nosemosis, cultivation, artificial infection

## Abstract

**Simple Summary:**

The microsporidium *Vairimorpha (Nosema) ceranae* is an emergent and highly virulent pathogen of the European honey bee. The study of this parasite and the development of new therapies is difficult due to the seasonality of the disease in nature, with a short period of availability of infected individuals for investigation. In this paper, we present and characterize an easy and convenient method for *V. ceranae* cultivation using artificially infected worker bees under laboratory conditions.

**Abstract:**

Nosemosis type C is a dangerous and widespread disease of the adult European honey bee *Apis mellifera* and is caused by the spore-forming intracellular parasite *Vairimorpha (Nosema) ceranae*. The search for new ways of therapy for this disease is complicated due to the seasonal availability of *V. ceranae*-infected insects as well as the lack of a developed system for the pathogen’s cultivation. By carrying out trials which used different infectious dosages of the parasite, spore storage protocols, host age, and incubation temperatures, we present a simple, safe, and efficient method of *V. ceranae* propagation in artificially infected worker bees in the laboratory. The method is based on feeding the groups of adult worker bees with microsporidian spores and insect maintenance in plastic bottles at 33 °C. The source of the spores originated from the cadavers of infected insects from the previous round of cultivation, in which the infective spores persist for up to six months. An analysis of five independent cultivation rounds involving more than 2500 bees showed that the proposed protocol exploiting the dosage of one million spores per bee yielded over 60 million *V. ceranae* spores per bee, and most of the spore samples can be isolated from living insects.

## 1. Introduction

First described in the Asian honey bee *Apis cerana* [1], the microsporidium *Vairimorpha (Nosema) ceranae* became an emergent and highly virulent pathogen in colonies of European honey bees *A. mellifera* around the world [2,3,4,5]. The infection caused by *V. ceranae*, nosemosis type C, leads to a decrease in adult density and honey production and is implicated in colony losses, at least in southern European countries [5,6]. For many years, this parasite was known as *Nosema ceranae*, but a recent phylogenetic revision of the genera of *Nosema* and *Vairimorpha* showed that this species should be assigned to the latter genus [7]. Here, we will use the current name of the species, although the traditional name is still commonly used.

The antibiotic fumagillin, isolated in 1949 from the fungus *Aspergillus fumigatus*, has been used to treat microsporidiosis in honey bees for more than 50 years [8,9]. This compound blocks the action of the *V. ceranae* methionine aminopeptidase-2 enzyme that is involved in the post-translational modification of the parasite’s proteins [10]. Although the treatment of infected hives with fumagillin significantly reduces the infection levels in the colonies and the risk of their destruction [11,12,13], the use of this substance is currently not authorized in Europe [13] due to its toxicity to mammals and the detection of antibiotic residues in apiculture products after treatment [14,15]. Along with the growing problems of the mass death of bees, thus impairing the pollination of a broad range of cultivated and wild plant species [4], this issue requires the search for new effective therapeutics and strategies to increase bee resistance to microsporidian pathogens. 

As opposed to other entomopathogenic microsporidia which are easily reproduced in their lab hosts, such as *Nosema bombycis* infecting the silkworm *Bombyx mori*, *Paranosema (Antonospora) locustae* from the migratory locust *Locusta migratoria*, and *P. grylli* from the bimaculate cricket *Gryllus bimaculatus*, the cultivation of bee pathogens is associated with serious difficulties. (1) The period for experimental work with naturally infected bees is limited. Although the infection of bees is increased during the winter, opening a hive to collect even a small insect sample from the colony during the cold period can kill it [16] (especially in northern countries with frosty winters), while the spring purgatory flight of bees is accompanied by a cleaning of their midgut and elimination of microsporidia from the hives. Thus, isolation of parasite spores from infected bees in temperate countries is most profitable in early spring [17]. (2) The insect may sting the personnel during experimental manipulations. (3) In contrast to the generalized microsporidian infection of the inner tissues of lepidopteran and orthopteran insects, *V. ceranae* growth is only observed in the epithelium of the bee midgut, which drastically reduces the spore yield from one insect. (4) Microsporidian spores isolated from insect intestines are contaminated with its contents. (5) Cultivation of honey bee microsporidia in vitro in insect cell cultures is a complicated procedure which does not allow for obtaining reliable yields of *V. ceranae* spores [18,19,20]. 

One of the ways to solve these problems is to isolate large amounts of parasite spores from infected insects in early spring and freeze them for consequent experiments [2,17]. Although the most efficient protocol for freezing *V. ceranae* spores was recommended among several techniques considered, their infectivity decreases by an order of magnitude after six months of storage. As noted above, another weak point of the mass spore isolation from naturally infected insects in early spring is sample contamination with the bee midgut contents, which is especially crucial for the inoculation of cell cultures grown under sterile conditions [18,19,20,21,22].

Here, we describe a simple, time-saving, labor-effective, accessible to every research group, and safe method for the propagation of *V. ceranae* in the laboratory. It provides researchers with infected bees suitable for physiological, biochemical and molecular analyses of parasites and infected host tissues during the entire period of their activity, and the method is suitable for infections of cell cultures with microsporidian spores as well. The results of the experiments, conducted to optimize the cultivation conditions, are also presented.

## 2. Materials and Methods

### 2.1. Obtaining V. ceranae Spores for Primary Infection

*V. ceranae* spores for initial inoculation were isolated from heavily infected live adult workers of honey bee *Apis mellifera carnica*, collected in early spring from a hive in a private apiary located in Russia, St. Petersburg, Vyakhtelevo village. The insects were euthanized by incubation at −20 °C for 10 min, and their intestines were extracted using sterile tweezers. The infection of bee midguts was diagnosed by bright field light microscopy using a Carl Zeiss Axio Lab A1 microscope (Carl Zeiss, Oberkochen, Germany). A PCR test was conducted using the method described earlier [23] and was applied to verify the specificity of infection and to sort out the cases of contamination by another microsporidium of honey bees, *V. apis*. The midguts of bees infected only with *V. ceranae* were homogenized in water using a glass homogenizer with a Teflon pestle. The homogenate was centrifuged at 900× *g* for 10 min, the pellet was resuspended in distilled water, and the number of spores was counted using a haemocytometer according to the previously described method [24].

### 2.2. Artificial Infection of Bees

**Figure 1:**
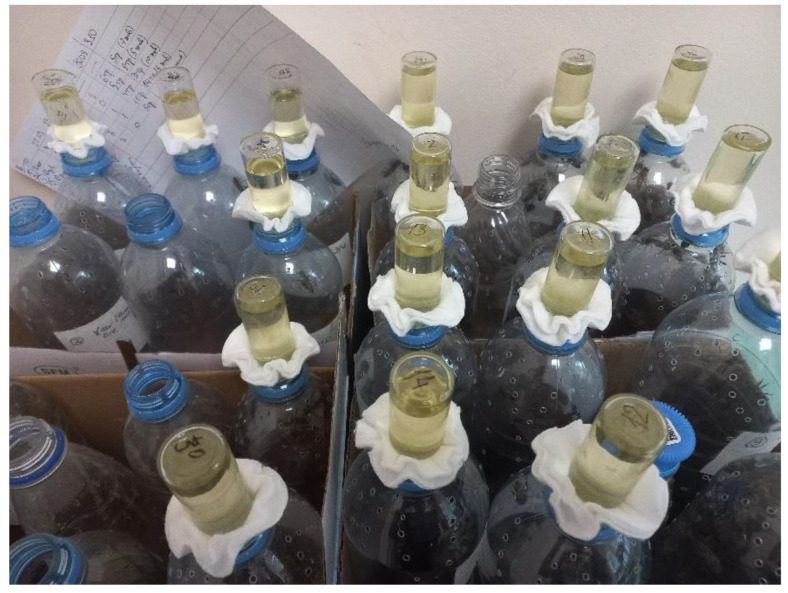
Laboratory maintenance of the worker honey bees for *Vairimorpha ceranae* cultivation. Insects were kept in plastic bottles with 40% sugar syrup poured into 10 mL glass vials closed with a cotton pad and inserted into the bottleneck.

Adult worker bees of *A. mellifera carnica* were obtained from the experimental apiary of the All-Russian Institute of Plant Protection (Russia, St. Petersburg, Pushkin). Insects of different ages (date of emergence) were collected from the central part of the hives where the younger bees are usually found [24]. To obtain newly emerged workers, a frame with a 21-day sealed brood was removed from the hive, placed in a closed plastic container, and incubated at 33 °C. In this case, insects which hatched during the day were sampled using a vacuum cleaner. A part of the sample was analyzed by microscopy to exclude natural infection by microsporidia. The collected bees were placed in perforated plastic bottles with a volume of 0.5 L using a funnel in groups of 20–30 individuals per bottle. The insects were fed with 40% sugar syrup poured into a 10 mL glass vials closed with a cotton pad, turned upside down, and inserted into the bottleneck (Figure 1). To infect the bees, a suspension containing either 0.3 or 1 million of *V. ceranae* spores per bee was applied to the surface of the cotton pad exposed to the insects.

### 2.3. Estimation of Insect Mortality and Infection Level

The bottles with bees were kept on laboratory tables at room temperature (about 25 °C) or in a closed box at 33 °C under natural light for 20–30 days. To feed the insects, the syrup in the glass vials was replenished upon consumption. For each infection, approximately 10–15% of the insects were not infected and were used as a control. Every 1–3 days, all the dead bees were taken from the bottles (by replacing the vial with a cap and flipping the bottle), and their amount was recorded. In experiments performed on small samples (less than 200 insects), the presence and intensity of *V. ceranae* infections were determined for each and every bee specimen according to the previously described method [24]. In experiments with a large number of individuals, a proportion of the sample was analyzed (15–60%) to provide data sufficient for statistical analysis. The live bees remaining by the end of experiment were anesthetized by incubation at 4 °C for 30 min and euthanized by decapitation. The intensity of microsporidian infection was estimated using the haemocytometer to count the number of spores per bee.

### 2.4. Obtaining Spores from Artificially Infected Bees and Their Storage Conditions

For re-infections of new samples of uninfected bees within one season, spores isolated from insect corpses from a previous infection were used. Estimation of infection levels and spore isolation were performed as above, except that the entire abdominal part was homogenized without an autopsy of the intestine to simplify the routine processing of the material. To resume the cultivation of *V. ceranae* in subsequent seasons of honey bee activity, parasite spores were either directly stored from November to March in the dried corpses of infected insects at room temperature or isolated from live insects and frozen at −80 °C in 10% glycerin solution [17]. In addition, infected bees that remained alive after 30 days of experimental observations were used to isolate purified spores for the inoculation of insect cells cultured in vitro [19,20,22,25].

### 2.5. Statistical Analysis

The data from the replicates within each variant of the experiments were pooled to increase the power of the statistical analysis. The differences in mortality scores on a given time point were estimated using Pearson’s chi-squared test [26] at 95% and 99% probability. Mean spore production, calculated as a proportion of the total number of spores observed in cadavers to the total number of bees that perished by the given time point, were compared between the variants using both the Kruskal–Wallis one-way analysis of variance on ranks and Mann–Whitney rank sum tests in SigmaPlot (Systat Software, San Jose, CA, USA).

### 2.6. Optimization of the Cultivation Conditions

Using the infection methods described above, we carried out the selection of the infectious dose (using 300 thousand or 1 million *V. ceranae* spores per 1 insect), the selection of the storage conditions for *V. ceranae* spores (using spores stored at −80 °C in a solution of 10% glycerol or in corpses of infected insects at room temperature), and the selection of the optimal temperature (incubation at room temperature or 33 °C) for keeping the bees during their artificial infection. Furthermore, the examination of the possibility of using newly emerged workers for infection was performed.

## 3. Results

### 3.1. Selection of the Infectious Dose of V. ceranae Spores for Artificial Infection of Bees

**Figure 2:**
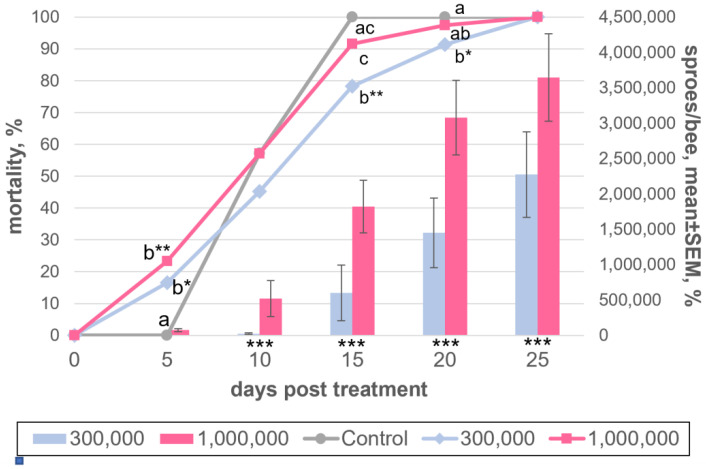
Dynamics of *Vairimorpha ceranae* development in adult honey bees experimentally infected with either 0.3 (“300,000”) or 1 million spores/bee (“1,000,000”) and maintained at 25 °C. Spores were isolated from dried honey bee cadavers stored at room temperature. The curves show cumulative insect mortality, and different letters indicate statistically significant differences at a given time point at *p* < 0.05 (*) or *p* < 0.01 (**), estimated using Pearson’s chi-square. Higher probability data are not indicated for clarity. The bars show the mean spore production, calculated as the ratio of the total number of spores observed in cadavers to the total number of bees that perished up to a given time point. The asterisks below bars indicate pairs of values that are significantly different from each other at *p* < 0.01 (**) or *p* < 0.001 (***) at a given time point, as inferred from the Kruskal–Wallis one-way analysis of variance on ranks and Mann–Whitney rank sum tests. The data which this scheme was created from are found in Table S1.

At the first stage of creating a *V. ceranae* cultivating system, it was necessary to determine the optimal infective dose of the parasite. To accomplish this, two samples of 115 and 154 worker bees of different ages were artificially infected using 300 thousand or 1 million *V. ceranae* spores per insect, isolated from bee corpses after primary infection, and stored for 1 month at room temperature. A sample of 28 uninfected bees kept under the same conditions was used as a control. Noticeably infected bees (with infection intensity levels that were higher than 1 million spores per insect) were detected as early on as on 10 dpi in the case of higher infection dose and only on 15 dpi at the lower dose. In all subsequent periods, the infection rates were significantly (*p* < 0.01) higher in the higher dose variant (Figure 2, Table S1). No infected bees were found in the control. In general, an increase in mortality was also observed with an increase in the dose of infection; however, high mortality level was also observed in the control, and in samples of this size it is not possible to significantly associate the mortality of bees with their infection with microsporidia.

### 3.2. Checking the Possibility of Using Newly Emerged Workers for V. ceranae Cultivation

**Figure 3:**
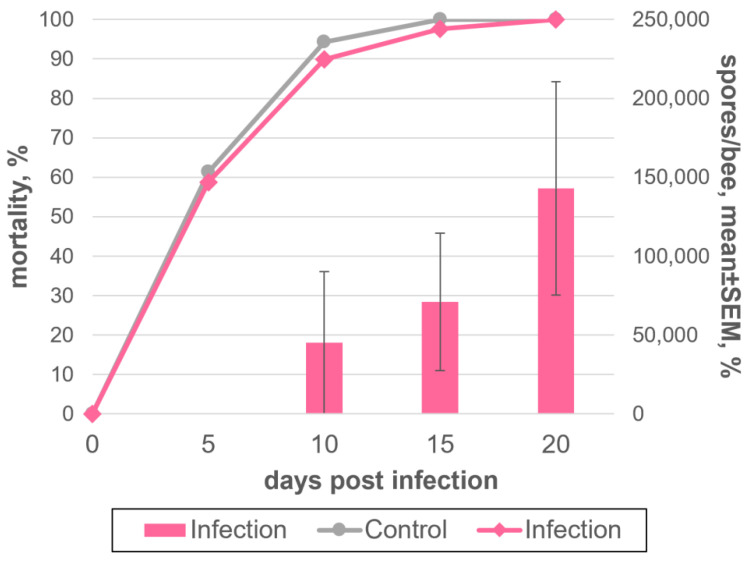
Dynamics of *Vairimorpha ceranae* development in newly emerged honey bee adults, maintained at 25 °C and experimentally infected with 1 million spores/bee; the spores were isolated from dried honey bee cadavers stored at room temperature. The indications are as in Figure 2. The data which this scheme was created from are found in Table S2.

Because it is not possible to accurately determine the age of worker bees when picking them directly from the hive, and because the sample may contain a certain proportion of older bees that will die due to senescence before development of the infection, we tried to use bees that emerged from the brood within one day (newly emerged workers) to unify the methodology. In two independent experiments, 349 insects were analyzed, 264 of which were infected with *V. ceranae*; the rest were kept under the same conditions without infection as a control. Because newborn bees usually do not feed on their own, adult bees of different ages (twenty-seven and eight individuals, respectively) were added to the samples, which could play the role of feeder bees. Bees were infected with 1 million *V. ceranae* spores isolated from dry insect corpses per one insect and kept at room temperature. As early on as on the 5 dpi (Figure 3, Table S2), we observed the death of more than half of all insects, both in the experiment and in the control. At 10 dpi, more than 90% of the bees had already died, and by the 15 dpi, only a few bees remained alive, the last of which died on the 19 dpi. The total yield of *V. ceranae* spores received on average per one insect for the entire experiment was only about 0.1 million, which is a tenth of the number of spores used for inoculation.

### 3.3. Selection of Storage Conditions for V. ceranae Spores

**Figure 4:**
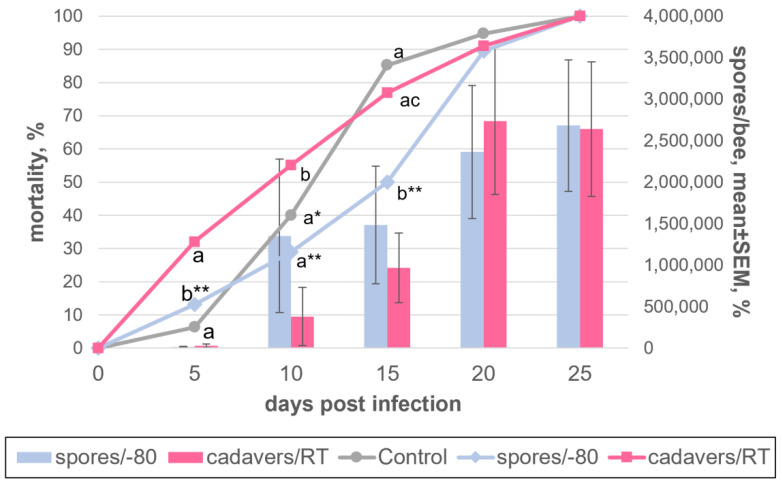
Dynamics of *Vairimorpha ceranae* development in honey bee adults, maintained at 25 °C and experimentally infected with 1 million spores/bee. Spores were either isolated from dried honey bee cadavers stored at room temperature (“cadavers/RT”) or isolated from live bees and frozen at −80 °C (“spores/−80”) prior to the experiments. The indications are as in Figure 2. The data which this scheme was created from are found in Table S3.

In order to determine the optimal method for storing *V. ceranae* spores during the cold period, when the bees are not active and there is no possibility to carry out experimental infection and maintain the parasite’s culture, we compared the most effective method of isolating and freezing spores described earlier [17] with the most simple approach implying the storage of dead infected bees at room temperature. For deep-freezing, the spores were isolated from live bees artificially infected in previous season at 20 dpi and stored for 5 months at −80 °C in a solution of 10% glycerol; corpses of infected insects were collected at 15–25 dpi and stored at room temperature for 5 months. At the beginning of the new season of bee activity, the spores were thawed or isolated from corpses after storage and used to infect two corresponding groups of 76 and 78 bees. Infection was carried out for 25 days at room temperature using an infection dose of one million spores per insect. A control sample of 94 uninfected bees was maintained under the same conditions. In both variants of infection, *V. ceranae* spores retained their invasiveness, the levels of infection of bees in all periods were similar, and the total production of spores per 1 bee during cultivation amounted to slightly more than 2.5 million spores (Figure 4, Table S3). No infected bees were found in the control.

### 3.4. Choosing the Optimal Temperature for Keeping Bees during Their Artificial Infection

**Figure 5:**
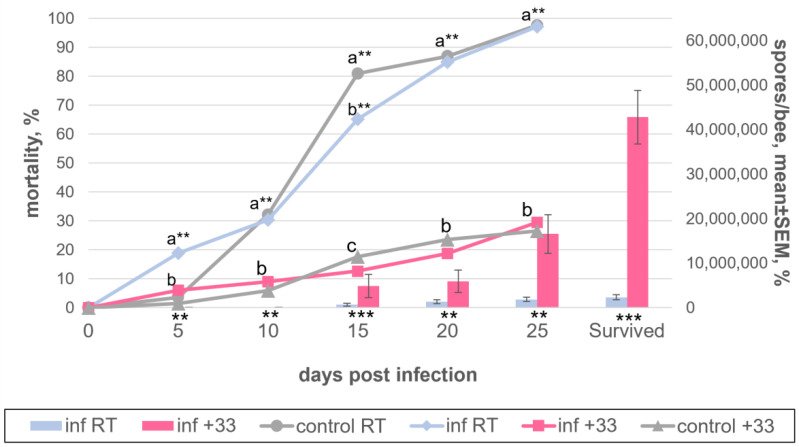
Dynamics of *Vairimorpha ceranae* development in honey bee adults, maintained either at +25 °C (“RT”) or at +33 °C (“+33”) and experimentally infected with 1 million spores/bee (“inf”). Spores were isolated from dried honey bee cadavers stored at room temperature. The indications are as in Figure 2. The data which this scheme was created from are found in Table S4.

To determine the optimal temperature for the maintenance of the infected bees, we inoculated 2 groups of 610 (plus 68 uninfected control individuals) and 569 (plus 84 control) bees of different ages with *V. ceranae*, using 1 million spores per individual bee. Spores were isolated from the corpses of infected insects and stored for 1 month at room temperature. The first group was kept at room temperature (about 25 °C) and the second in a box with a constant temperature of 33 °C for 25 days. A comparative analysis of the groups showed that when the insects were kept at 33 °C, their mortality significantly decreased. In fact, at 25 dpi, only about a quarter of the bee sample died, while at room temperature, almost all insects had already died (Figure 5, Table S4). At each time point, starting from 15 dpi, the level of infection of bees was significantly higher in the second group, and the total yield of infection for 25 days was almost 20 times higher and amounted to 42.8 million spores per insect, as opposed to 2.3 million spores when kept at room temperature. No infected bees were found in the control.

### 3.5. Summary Statistics of Mortality and Infection Level of Artificially Infected Bees under the Optimal Variant of the Microsporidium V. ceranae Cultivation

**Figure 6:**
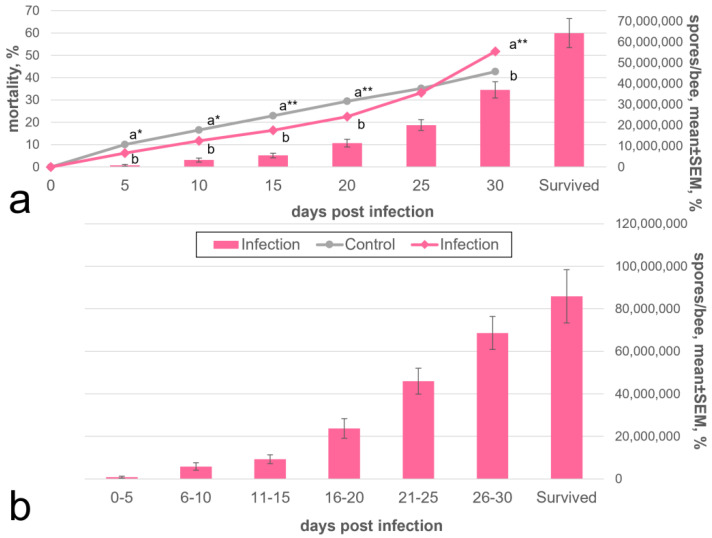
Dynamics of *Vairimorpha ceranae* development in adult honey bees, maintained at 33 °C and experimentally infected with 1 million spores/bee. Spores were isolated from dried honey bee cadavers stored at room temperature. The mean number of spores per bee was calculated using estimates of the total amount of bees from the beginning of the experiment (**a**) or within a given time interval (**b**). The indications are as in Figure 2. The data which this scheme was created from are found in Table S5.

After the optimal cultivation conditions for *V. ceranae* had been chosen, we performed a cultivation of the parasite for 2 years in several rounds of artificial infection for each season of bee activity. The first infection round was run in the spring using *V. ceranae* spores from the corpses of infected insects obtained in the previous year and kept at room temperature. Subsequently, for infections during one season, spores from the corpses of infected insects obtained from the previous infection were used. Each cultivation cycle was carried out at 33 °C for 30 days, and the infectious dose was 1 million *V. ceranae* spores per bee. The cultivation cycle series was ended by mid-autumn, and dead infected bees were stored until the following series. Insect mortality and infection rates significantly varied between different cycles (up to 30% difference, data not shown). This is most likely because the conditions under which the worker bees were collected from the hives were subject to change. First of all, the seasonal fluctuations of weather governing the temperature and humidity in the apiary has a very significant impact on bees’ fitness in the experiments [17,27,28]. Within this framework, we did not focus on sufficient data collection to demonstrate the respective patterns but preferred to provide the overall dynamics of bee mortality and infection levels summarized for the 5 cultivation cycles (performed in spring, summer and autumn of 2 different seasons) with a total of 2251 infected and 279 uninfected (control) individuals. Interestingly, at 5 dpi, the mortality of the control bees was slightly higher than that of the infected bees (Figure 6, Table S5). Then, up to the 20th day, mortality increased relatively equally and linearly, by 5–7% of the sample every 5 days, after which the mortality of infected bees began to increase much faster than the control, which is probably associated with the onset of bee death from nosemosis. An insignificant level of infestation of bees, approximately 0.8 million spores per individual, was already observed by 5 dpi, which then significantly increased at each time interval (Figure 6). On average, about 64 million *V. ceranae* spores were obtained for each of the 2251 bees during the 30 days of cultivation, with most of the spores being isolated from the 1086 insects that survived by the end of the cultivation period and an average infection rate of about 86 million spores per bee (Figure 6). No infected bees were found in the control.

## 4. Discussion

Based on the conducted studies, we propose the following optimal method for the propagation of the microsporidium *V. ceranae* under laboratory conditions. During the season of bee activity, remove healthy workers from the center of the hive and place them in 0.5 L plastic bottles (20–30 bees per bottle). To feed the insects, use 10 mL cotton-topped glass vials inserted into the bottle necks. To inoculate the bees, apply a *V. ceranae* spore suspension to cotton caps at the dosage of 1 million spores per bee and expose it to the workers in the bottle. For the first cultivation cycle, use the spores isolated from naturally infected living insects after checking for *V. apis* contamination with microscopy and PCR; for the subsequent rounds, use spores from the cadavers of infected bees obtained during the last cultivation round. Infected bees should be maintained at 33 °C; perished bees should be removed, and the sugar syrup should be renewed every 2–3 days. The period between the 20th and 30th day of infection is optimal for spore isolation from the infected insects. During this time, most of the bees remain viable and highly infected. To repeat artificial infection in the next season, it is sufficient to preserve the dry corpses of infected insects, in which the infectivity of parasite spores persists for up to six months, or to freeze the spores according to the described methods [17].

High mortality levels of bees at room temperature and the need to maintain the infected bees at 30–33 °C is widely acknowledged [27,28]. We, however, planned to design the simplest protocol using as little equipment as possible; thus, we performed the first experiments at room temperature. However, there were such drastic differences in the bee mortality levels and spore yields depending upon the temperature (Figure 5) that all further experiments were carried out at 33 °C.

Artificial infection of *A. mellifera* worker bees by *V. ceranae* spores has already been described many times in the literature [17,29,30,31,32,33]. In most cases, the technique used an implied individual feeding of each adult worker honey bee with a suspension of *V. ceranae* spores. This made it possible to adjust the infectious dosage in each bee while effectively inoculating the insects. The numbers vary between studies, but on average, doses of several tens of thousands of spores per bee were sufficient for achieving almost 100% infection of bees, usually resulting in bee death on 5–10 days after infection. Obviously, the effectiveness of such methods of infection in terms of the ratio of infecting dose and the degree of infection of insects significantly exceeds that proposed by us. In addition, in our case, it is impossible to control the exact dose for each bee, as the infection randomly occurs. Only a certain part of these spores enters the digestive tract of bees, which is different for each individual. In addition, it is important to note that for individual infection, fresh spores were isolated from living infected insects. As shown, the infectivity of such spores is almost ten times higher than, for example, when frozen and stored at −80 °C [17]. In our experiments for group infection, we used spores from insect corpses. Because their infectivity was comparable to frozen ones (Figure 4), the need to use a high dose for infection is quite expected. There are also a few papers that describe group infection of bees with spore doses similar to those used for individual feeding [34,35]. However, the degree of infection of insects in these experiments was significantly lower than that obtained here.

Nevertheless, in our opinion, a relatively high dose of spores and a low infection rate in the first dpi is not a drawback of our method. The main goal of *V. ceranae* cultivation in this study was to ensure the constant availability of live microsporidia-infected bees in the laboratory for the isolation of fresh parasite spores needed for experiments related, for example, to the artificial infection of cell cultures. In this case, an extremely high degree of infection of bees was not needed because the insects will only survive a few days. Thus, we did not try to increase the infectious dose in our experiments. In addition, it is worth noting that after a 30-day cultivation round, we received more than 60 million spores per infected bee (Figure 6), and it is not a problem to use some of them for the next infection cycle.

**Figure 7:**
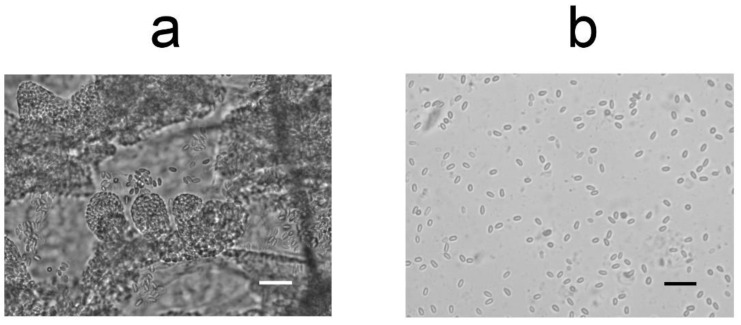
Light microscopy of artificially infected worker bees. (**a**) Segment of infected bee intestine after 30 days of *V. ceranae* cultivation; (**b**) homogenized infected bee intestine after 30 days of *V. ceranae* cultivation. Scale bars: 20 mkm.

We believe that one of the important positive properties of the proposed method is its safety. Direct interaction with active insects is carried out only by an apiary worker, who transfers the bees from the hive to the bottles. Inoculation and feeding of bees occur without the researcher’s contact with active insects. When live infected bees are to be used, they are immobilized by chilling or freezing directly in the bottles and can be processed without the risk of being stung. Another positive feature of the technique is its simplicity, accessibility, the minimum amount of required equipment, and the low cost of all consumables, such as ordinary plastic drinking water bottles, which can be used several times after cleaning and sterilization. Another advantage of the proposed technique is that during long-term feeding with sugar syrup, the microsporidium-infected bee midguts are completely cleared of pollen and other contents in as many as 50% of the insect specimens (Figure 7). This greatly facilitates the purification of spores from the insect midgut, as the filtration through a nylon filter, required when using bees from hives for in vitro experiments [18,20], is avoided, as shown in our experiments with the inoculation of insect cell lines [19,20,22,25].

As a limitation of our method, we can note the impossibility of the effective infection of newly emerged worker bees selected within one day after hatching from the brood. We attribute this negative result to the fact that such individuals cannot yet fully feed on sugar syrup on their own, which leads to their mass death in a short time and an almost absent level of infection, even when a small number of adults of different ages that are potentially able to act as breadwinners were added to the bottles with the newly emerged bees. Nevertheless, the advantages of the proposed technique significantly outweigh this “disadvantage”.

## 5. Conclusions

Thus, the proposed method for cultivation of *V. ceranae* in artificially infected worker bees allows us: (1) to keep insects in the laboratory for a long time and safely infect them with the parasite spores; (2) to propagate the microsporidium and obtain a large amount of spores forming in the epithelial cells of the infected bee midguts; (3) to effectively remove the contents of the intestine and obtain clear infected tissues and parasite spores; and (4) to maintain the invasiveness of spores between artificial infestations and during the cold season.

## Data Availability

The raw data used for the creation of the schemes in this article are in the tables in Supplementary Materials.

## Supplementary Materials

The following supporting information can be downloaded at: https://www.mdpi.com/article/10.3390/insects13121092/s1, Table S1: Dynamics of *Vairimorpha ceranae* development in adult honey bees experimentally infected with either 0.3 or 1 million spores/bee and maintained at 25 °C; Table S2: Dynamics of *Vairimorpha ceranae* development in newly emerged honey bee adults, maintained at 25 °C and experimentally infected with 1 million spores/bee. The spores were isolated from dried honey bee cadavers stored at room temperature; Table S3: Dynamics of *Vairimorpha ceranae* development in honey bee adults, maintained at 25 °C and experimentally infected with 1 million spores/bee. Spores were either isolated from dried honey bee cadavers stored at room temperature or isolated from live bees and frozen at −80 °C prior to the experiments; Table S4: Dynamics of *Vairimorpha ceranae* development in honey bee adults, maintained at either RT or at +33 °C and experimentally infected with 1 million spores/bee. Spores were isolated from dried honey bee cadavers stored at room temperature; Table S5: Dynamics of *Vairimorpha ceranae* development in adult honey bees maintained at 33 °C and experimentally infected with 1 million spores/bee. Spores were isolated from dried honey bee cadavers stored at room temperature.
